# Climate Extremes in Consecutive Years Impacted the Number and Fate of Duck Nests on Great Salt Lake Marshes

**DOI:** 10.1002/ece3.70630

**Published:** 2024-12-06

**Authors:** Michael R. Conover, Mark E. Bell

**Affiliations:** ^1^ Ecology Center and Department of Wildland Resources Utah State University Logan Utah USA; ^2^ Department of Fisheries and Wildlife Michigan State University East Lansing Michigan USA

**Keywords:** cinnamon teal, daily survival rates, depredated nests, gadwall, incubation‐initiation date, mallard, nest densities, nest success, raccoon, striped skunk

## Abstract

The number of ground‐nesting ducks in the marshes of Great Salt Lake (GSL), Utah has drastically decreased in the past few decades. One potential cause for this decline is the increase in climate extremes caused by global warming. From 2019 through 2023, GSL marshes experienced 1 year of historic spring rainfall (2019), 2 years of historic droughts (2021 and 2022), and 1 year of record snowfall (2023). We used this time period to test the hypothesis that climate extremes impact both the number of duck nests and their fate (i.e., successful, depredated, or abandoned). We counted 563 nests of cinnamon teal (*Spatula cyanoptera*), 168 mallards ( *Anas platyrhynchos* ), and 220 gadwalls *(Mareca strepera*). Nest numbers varied among years and were positively correlated with the amount of spring rainfall (April and May). Clutch sizes differed among years and were lowest during the drought years. Raccoons ( *Procyon lotor* ) and striped skunks ( *Mephitis mephitis* ) were the major predators of nests. The percentage of all duck nests that were depredated varied among years and increased from 40% and 46% during the 2 wet years to 75% and 90% during the 2 drought years. The percentage of nests that were successful varied among years and were highest during the wet years. The yearly percentage of successful nests was negatively correlated with the abundance of all predators and positively correlated with snowfall because few skunks and raccoons survived the winter of 2023 with its heavy snowfall. Daily survival rates ($\bar{x}$ = 0.93), were similar among duck species, but varied among years; DSRs were lowest during the drought years (0.86 and 0.92) and highest during the wet years (0.96 for both years). Our results suggest that climate extremes will have an adverse impact on both the number of duck nests and the percentage of them that are successful.

## Graphical abstract

A six‐year period of climate extreme from 2019 to 2023 had a significant impact on the number of nesting ducks, clutch sizes, and the proportion of nests that were success. Most failed nests were depredated by raccoons and striped skunks.

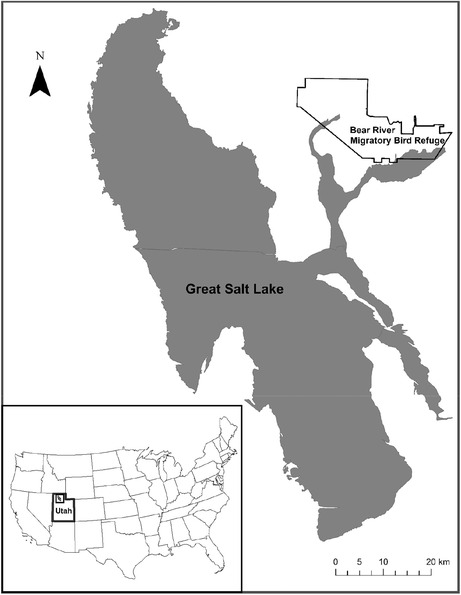

## 1 Introduction

Historically, tens of thousands of cinnamon teal (*Spatula cyanoptera*), mallard ( *Anas platyrhynchos* ), and gadwall (*Mareca strepera*) nested on Great Salt Lake (GSL) marshes, United States. These marshes were the heart of the breeding range for cinnamon teal, with over half (150,000 out of 300,000) nesting along GSL prior to 1980 (Bellrose 1980). Unfortunately, aerial surveys of breeding waterfowl in Utah from 1990 to 2010 counted an average of 22,000 breeding pairs of cinnamon teal along GSL (Kear 2005; Baldassarre 2014). More recently, Olson (2016) reported only 10,000 nesting pairs of cinnamon teal throughout Utah.

A potential reason for the decline in the number of nesting ducks is that both raccoons ( *Procyon lotor* ) and red foxes ( *Vulpes vulpes* ) expanded their range northward in the 1980s to include northern Utah and GSL marshes (West 2002; Frey and Conover 2006). It is not clear what allowed raccoons and red foxes to expand their range northward into northern Utah, but climate change from global warming is a possibility. Before 1980, striped skunks ( *Mephitis mephitis* ) were the only major predator of duck nests in GSL marshes (Crabtree, Broome, and Wolfe 1989), and duck nests were so numerous that only a small percentage of them were depredated (Crabtree and Wolfe 1988; Crabtree, Broome, and Wolfe 1989). Since then, populations of skunks, raccoons, and foxes in GSL marshes have increased to the point where few duck nests survive (Conover and Bell 2020; Bell 2022; Bell and Conover 2023b).

Climate change is producing more dramatic fluctuations in weather patterns across the globe, including the western United States, where extreme weather events are becoming more intense and frequent (Strzepek et al. 2010; Dettinger, Udall, and Georgakakos 2015; Lute, Abatzoglou, and Hegewisch 2015) and may be directly impacting the number and fate of duck nests. This trend was strikingly evident on GSL marshes from 2019 to 2023. During 2019, rainfall during the spring reached historic levels. This was followed by historic droughts during 2021 and 2022 when the water level of the GSL dropped to historic lows. Those drought years were followed in 2023 with record snow levels in northern Utah and in the GSL marshes themselves. There is concern that severe droughts and floods have a direct impact on ducks nesting around GSL, which are already in a precarious position. In this paper, we tested the hypothesis that recent floods and droughts are related to the number of ducks nesting around GSL and their success.

## 2 Study Area

Utah is the second driest state in the United States and is classified as a cold desert biome. Directly to the west of Great Salt Lake is the Great Salt Lake Desert, which extends westward into Nevada. Great Salt Lake has an elevation of 1283 m above sea level and lies within the Great Basin, so named because there is no outlet to the sea. Most precipitation in the GSL watershed occurs during the winter as snow, most of which falls on the Wasatch and Uinta mountain ranges (elevations up to 4100 m). This snow melts during the spring and summer, and rivers carry the water to GSL.

**FIGURE 1:**
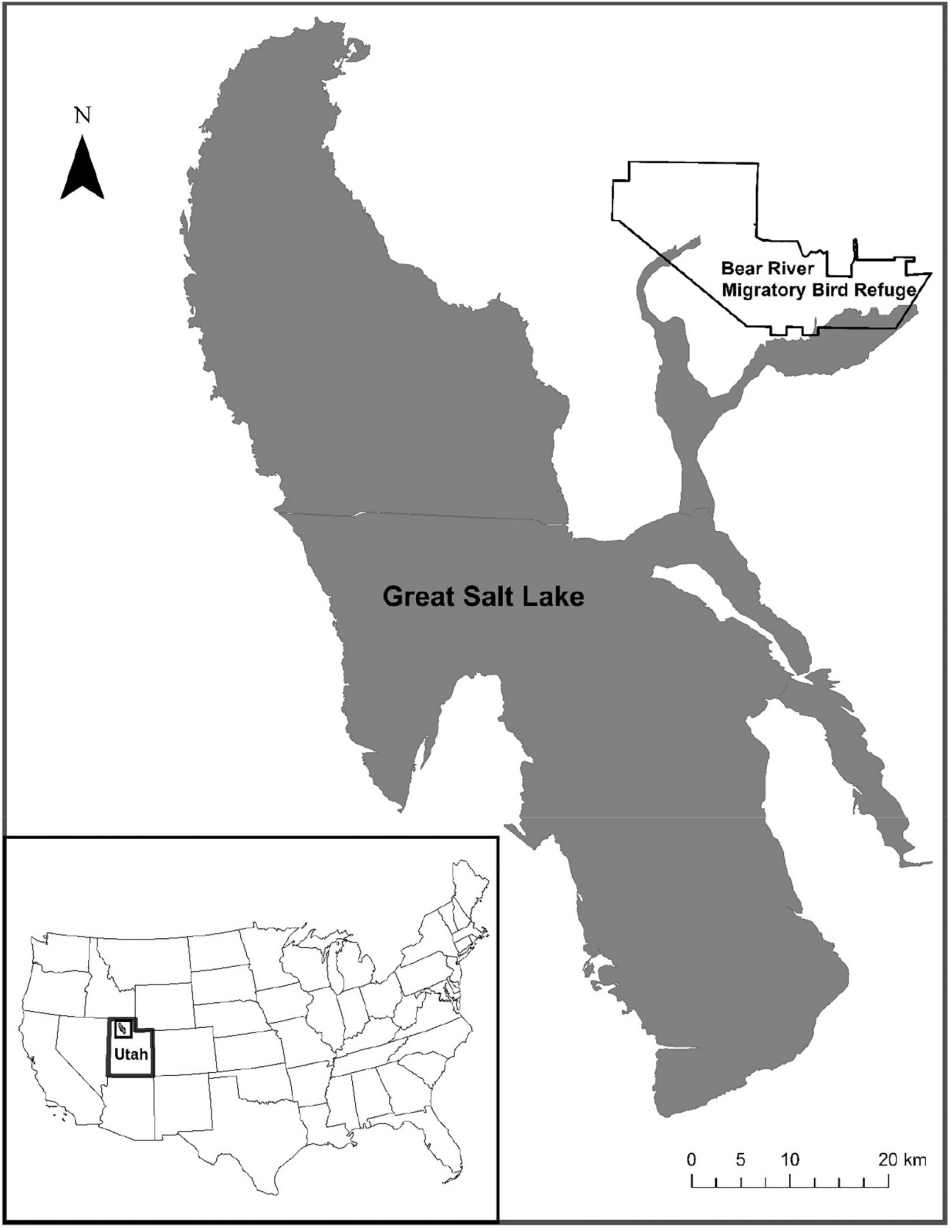
Map showing the location of the US Bear River Migratory Bird Refuge.

**FIGURE 2:**
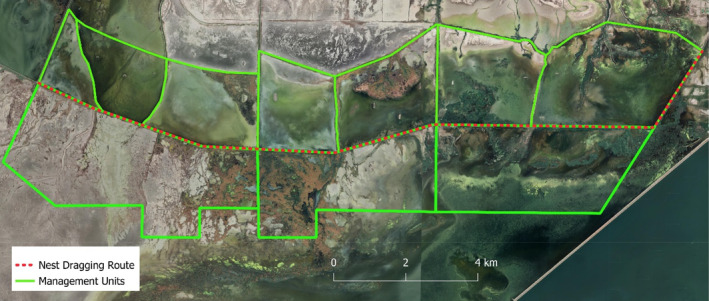
Map of the US Bear River Migratory Bird Refuge showing the transect where we searched for duck nests and the 10 impoundments that border our transect.

Our study was conducted in the US Bear River Migratory Bird Refuge (BRMBR), which was created on the vast delta where the Bear River flowed into the eastern side of GSL (Figures 1 and 2). Low dams were built across the delta to provide a series of shallow (< 2 m deep) impoundments, which were managed for ducks and other waterbirds. These impoundments were adjacent to each other, allowing the maximum amount of land to be flooded. As a result, dams were the only dry land to provide nesting habitat for dabbling ducks during wet springs. Dams were constructed so that both sides of the dams had a gentle slope from the water's edge up to the road, which was built on the dam's crest. These sloping sides were covered by grass and forbs and provided nesting habitat for ducks, other waterfowl, and shorebirds; trees did not occur on the dams or in the marshes. Historically, ducks nested within the shallower parts of impoundments (Williams and Marshall 1938), but few ducks nested there during recent years, owing to invasive phragmites (Phragmites australis) overgrowing these areas (M. Conover, unpublished study). Our study was limited to the section of BRMBR that was closed to the public because this was where most ducks nested. Duck species we found nesting in BRMBR included cinnamon teal, mallard, and gadwall.

## 3 Methods

We searched both sides of 20 kms of dams within BRMBR for duck nests every 2 weeks from May until August from 2019 to 2023. We searched one side of the dam as we drove along the road on the crest of the dam and searched the opposite side as we came back the opposite direction towards the starting point. Potential nesting habitat included the sides of the dam where forbs and grass cover existed and ducks nested. We excluded the road in the center because no ducks nested on the road. In total, we searched 0.28 km^2^ of nesting habitat each year, and the same stretch of dam was searched every year.

**FIGURE 3:**
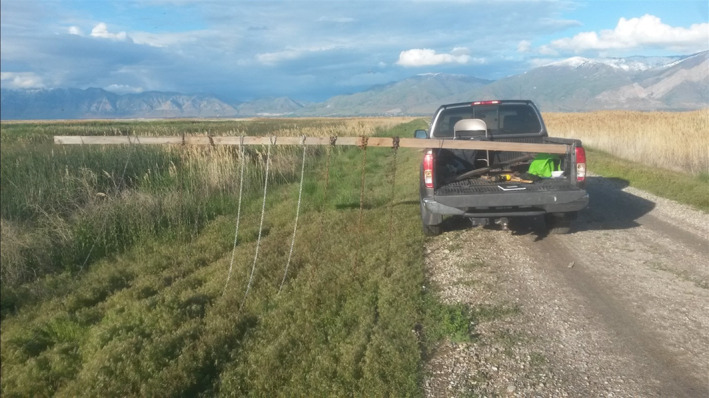
Photo of the apparatus we used for our modified chain‐dragging method. A boom was constructed out of lumber (3.8 cm in height and 8.9 cm in width, commonly referred to as 2 by 4 s), which extended out the driver's side of a pickup bed 4–6 m. The boom length was adjusted to accommodate the width of the dam being searched. Chains were attached to the boom and spaced approximately 25 cm apart. These chains were 8–12 m in length and dragged straight behind the boom as the truck drove along the dam at 10–15 km/h.

We conducted nest searches between 09:00 and 15:00 h as recommended by Gloutney et al. (1993). Nests were located using a modified chain‐dragging method (Klett et al. 1986). A boom was constructed out of lumber (3.8 cm in height and 8.9 cm in width, commonly referred to as 2 by 4 s), which extended out one side of a pickup bed 4–6 m (Figure 3). The boom's length was adjusted to accommodate the width of the dam being searched. Chains were attached to the boom and spaced approximately 25 cm apart. These chains were 8–12 m in length and dragged straight behind the boom as the truck drove along the dam at 10–15 km/h. The chains flowed through the vegetation without disturbing vegetation or nests. As the chains passed over or close to the nests, the incubating hen would flush, allowing an observer in the back of the pickup to locate the nest and identify the species of duck by its plumage. The eggs were lower than the top of the nest bowl so the chain did not touch them; only one egg was crushed by the chain during this study.

Once we located a nest, one person approached the nest to record its GPS location. Rubber boots were worn to minimize cues to predators, such as odor. We kept our time at nests to less than 5 min to minimize our disturbance and to allow the hen to return quickly to its nest. We gave each nest a unique number and marked it by placing two survey flags on the opposite side of the road to aid in relocating the nest for monitoring purposes. A small marker was placed inconspicuously on the ground under the vegetation next to the nest, which assisted in relocating the nest after nesting was completed. At each nest, we recorded clutch size and determined the age of the nest by randomly selecting one egg from it and determining the age of its embryo using the floatation method (Brua and Machin 2000). We decided to use this method after comparing the floatation method with the candling method (Weller 1956; Montgomery, Burke, and Byers 1978). We had our technicians age the same duck egg on the same day using both methods; we found that variation in age estimations were less using the flotation method. The incubation‐initiation date was the day when the hen started incubating the nest and the embryo started to grow. We determined the incubation initiation day for each nest by noting the ordinal day when the nest was first discovered and subtracting from that ordinal day and age of the embryo, which was measured on the same day that the nest was discovered.

Each nest was revisited at least every 2 weeks, at the same time the dam was being searched for new nests. During each visit, we measured clutch size and determined if the nest was still being incubated. The latter was determined by flushing a hen from the nest or noting that the eggs were warm. We determined the fate of each nest as depredated, abandoned, or successful by observing egg remains (Klett et al. 1986). Missing or scattered eggs or broken eggshells with membranes attached to the shell indicated a depredated nest. Eggshells with membranes separated from the shell characterized successful nests. A nest was considered successful if ≥1 egg had hatched. A nest was considered abandoned if the eggs appeared undisturbed by a predator and were cold and unattended by a hen for two consecutive visits to the nest.

Game cameras (Cuddeback 20 Megapixel IR, Cuddeback, De Pere, Wisconsin) were installed at approximately 20% of the nests annually to observe the nest, using the methods of Croston et al. (2018), Krüger et al. (2018), and Blythe and Boyce (2020). Camera images were utilized to confirm a nest's fate and to identify the species of predator that depredated the nest (Bell and Conover 2023c). Cameras were placed approximately 0.5 m away from the nest. Height of cameras was adjusted, setting it so that camera was below the surrounding vegetation. New batteries and a new SD memory card were placed in each camera during each nest search. The cameras were set to just take still photos, and a timestamp was printed on each photo.

We analyzed data annually first for each duck species separately and then with all species combined. Our dependent variables were (1) abundance of duck nests, (2) clutch size, (3) incubation‐initiation date, (4) number of nests that were depredated, abandoned, or successful; and (5) daily survival rates.

The variable, abundance of duck nests, was a count of all nests we found during a single year that was corrected by using the variability in daily survival rates (DSR) among years. This was achieved by dividing the number of nests located during a specific year by that year's DSR raised to the mean embryo age in days when the nest was first discovered (McPherson et al. 2003). If we had not done this, then nest abundance would be lower in years with low a DSR because a higher percentage of nests would have been depredated before we located them.

Clutch size was the largest number of eggs found in a nest during our nest searches. The variable, incubation‐initiation day, was the ordinal day when a nest was first incubated and embryos began to develop (Baldassarre 2014). It was determined by recording the ordinal day when the nest was first located and then subtracting from that ordinal day the age of an embryo from that nest; the latter was measured on the same day that we first located the nest.

We used the timestamp on the first photo of a predator at a nest to determine the exact date when the nest was depredated. For successful nests, we could determine the exact day of hatching when we visited a nest and found ducklings inside it or when a photo from a nest camera showed ducklings in the nest. When a nest was depredated or hatched between two nest visits and there was no photo of a predator at the nest, we assumed that the event occurred at the midpoint of the last visit before the nest's fate was determined and the first visit after that. A nest's fate was determined when the nest was depredated, abandoned, or was successful.

Using the fate of nests under observation to determine nest success is called the Apparent Method. This method provided useful data for comparative purposes, and we used it to determine the fate of individual nests. To determine the percentage of nests that were depredated, abandoned, or successful, we divided these number of nests in each category by the total number of nests each year where we were able to determine their fate. We were unable to determine the fate of a few nests because we failed to relocate some nests during later searches. For this reason, the number of nests with known fate was lower than the total number of nests we located.

The Apparent Method overestimates the actual percentage of nests that were successful because this method fails to account for nests that were depredated before we located the nest. A better method to assess the percentage of nests that are successful is the Mayfield Method, which can account for nests that were depredated prior to being located. The Mayfield Method is based on the probability of a nest surviving 1 day, which is called the daily survival rate (DSR). This can be calculated by dividing the number of successful nests by the total number of days that all nests were under observation and exposed to predators and other threats (called exposure days; Mayfield 1961, 1975; Johnson 1979; Klett and Johnson 1982). The probability of a duck nest surviving the entire incubation period (i.e., nest survival rate) can be determined by exponentiating a species' DSR by the number of days in the incubation period for that species.

Our independent variables included year, annual snowfall, spring precipitation, raccoon abundance, skunk abundance, all‐predator abundance, and number of skunks and raccoons removed annually from BRMBR. We measured annual snowfall because the number of predators in the BRMBR during the nest incubation period may have been affected by snow levels during the prior winter. Spring precipitation was measured from April 1 to May 31 because the snow in BRMBR had melted by then, plant growth had begun, and ducks started selecting nest sites in early June. We used this time period rather than the entire incubation period because we wanted to assess conditions when the ducks were deciding if and where to nest. These variables were measured at the weather station closest to BRMBR that had the required data and was at a similar elevation (within 20 m) as BRMBR. Yearly snowfall was measured at Brigham City Municipal Airport, which was 7 km north of BRMBR (Extreme Weather Watch 2023). We measured spring precipitation at Hinckley Airport in Ogden, which was 30 km south of BRMBR (Weather Underground 2023).

Abundances of raccoons, striped skunks, and total predators were used as both independent and dependent variables to ascertain the impact of these predators on the other dependent variables. We measured predator abundance by installing the same game cameras, which were also used for nest monitoring, at each of the 12 bridges along the dams where we conducted our nest searches. We monitored predators at bridges during a 21‐day period immediately prior to the start of nest searches. We wanted to keep human activity on the dams to a minimum when ducks were laying eggs or incubating them, so we did not monitor predator activity during this period. Bridge cameras recorded the number of raccoons, skunks, and all predators that crossed any bridge every night over the three‐week period during 2020, 2021, and 2023. Sightings of the same predator species at the same bridge that occurred within 2 min of each other were considered the same individual and were only counted once. Predator abundance was a measure of the mean number of predators that were photographed nightly crossing a single bridge during a single night over a 21‐day period in May right before ducks started incubating their nests. There were 12 bridges along our transect, and one camera was positioned at each bridge. Our predator counts provided an index of the relative abundance of predators; these counts were not a measure of the number of predators in the population or predator densities because we could not individually recognize a predator.

The BRMBR trapped mammalian predators during several years of this study. The refuge provided us with data on the years when predator removal took place and the number of raccoons and skunks removed each year. We used the numbers of skunks and raccoons removed annually as independent variables.

All statistical tests were conducted using Number Cruncher (NCSS, Provo, Utah). We used Pearson's chi‐square test (Little and Hills 1978) to compare the number of nests each year to the expected number of nests if they were evenly distributed among years. The expected value was the sum of nests during all years divided by five (the number of years when data were collected). Expected values used in this study are shown in the tables. We considered a result to be statistically significant if *p* < 0.05 in this and all other statistical tests used in this study.

We used the one‐way analysis of variance test (Little and Hills 1978) to determine if clutch size or the ordinal day of incubation initiation varied over years. These two dependent variables were real numbers and were normally distributed. We conducted three Pearson's chi‐square tests to determine if the number of nests that were depredated versus not depredated varied among years, if the number of nests that were abandoned versus not abandoned varied among years, and if the number of nests that were successful versus not successful varied among years.

We modeled the DSR of nests using the nest survival analysis (Dinsmore, White, and Knopf 2002) in program MARK (White and Cooch 2001). We included each year and species as parameters in our model to test for any variation in DSRs among years or species. We tested whether DSRs varied among years by using estimates for 2019 as the reference year and comparing estimates from other years to it. Likewise, we used estimates for cinnamon teal as the reference species and compared estimates of gadwall and mallard to it. We inspected the model output and considered parameters with an estimated 95% confidence interval that did not cross zero to be significant in impacting DSRs. We then retrieved the DSR estimates from program MARK for each parameter that was significant.

We used the Pearson's chi square test to assess if predator numbers varied among years by counting the number of predators that were photographed each year crossing a bridge and comparing this to the expected number, which was the mean number of predators photographed during 2020, 2021, and 2023 divided by the number of years (3). Likewise, we determined if the number of skunks or raccoons removed each year varied among years by comparing the numbers removed each year with the mean number for all years combined during 2019 through 2023.

We used a Pearson correlation analysis (Little and Hills 1978) to determine if annual variation in any of our dependent variables was correlated with annual variation in any of our independent variables. Prior to these analyses, we also conducted a Pearson correlation analysis with all combinations of independent variables to test for collinearity. We used a *r* > 0.60 as a measure of collinearity among independent variables (Dormann et al. 2013). When independent variables were collinear, we included only one in our model. We decided to include the one that the scientific literature indicated was the most important for nest‐site selection by ducks. Skunk abundance and raccoon abundance were collinear (*r* = 0.87), and both were collinear with the abundance of all predators. Nevertheless, we decided to test all three measures of predator abundance because they served as both dependent and independent variables.

## 4 Results

The model output from program MARK showed that the drought years (2021 and 2022) significantly impacted DSRs because the 95% confidence intervals for those 2 years did not cross zero. In contrast, duck species did not impact DSRs (Table 1). We retrieved the DSRs from MARK for each year, finding a DSR (± SD) of 0.964 ± 0.05 for 2019, 0.961 ± 0.04 for 2020, 0.864 ± 0.16 for 2021, 0.923 ± 0.08 for 2022, and 0.964 ± 0.05 for 2023. DSRs were lowest during the dry years (2021 and 2022).

**TABLE 1:** Model results from nest survival analysis performed in program Mark (White and Cooch 2001; Dinsmore, White, and Knopf 2002). Results for each parameter along with its 95% confidence interval (CI) are shown. The reference variables are 2019 for year and cinnamon teal for species. We included each year and species as parameters in our model to test for any variation in daily survival rates among years or species. Parameters with a 95% CI that did not cross zero had a significant impact on daily survival rates (DSRs) from the reference variable. Years 2021 and 2022 differed in DSR from 2019. Neither of the species varied in DSRs from cinnamon teal. Data were collected at US Bear River Migratory Bird Refuge, Utah.

Parameters	Estimate	95% CI
Intercept	3.245	3.018 to 3.471
2020	−0.068	−0.415 to 0.279
2021	−1.352	−1.793 to −0.910
2022	−0.739	−1.134 to −0.344
2023	−0.012	−0.341 to 0.317
Gadwall	0.214	−1.02 to 0.531
Mallard	−0.134	−0.456 to 0.188

From 2019 through 2023, we located 703 duck nests, but after we corrected the number of nests we found to account for nests that were depredated before we found them, the actual number of nests was 951 (563 cinnamon teal, 168 mallards, and 220 gadwall). Nest numbers of all duck species combined varied significantly among years; nest numbers (293) during the wet year of 2019 were more than twice as high in as any other year (Table 2). When each duck species was tested separately, nest numbers varied among years for cinnamon teal, mallard, and gadwall; each followed the same pattern with nest numbers increasing during wet years (2019 and 2023). The annual variation in nest abundance between the highest and lowest nesting numbers was 10:1 for cinnamon teal, 4:1 for mallard, and 14:1 for gadwall. Nest densities (number/km^2^) across all years were 409 for cinnamon teal, 126 for mallard, 159 for gadwall, and 694 for all duck species (Table 3).

**TABLE 2:** Pearson's Chi‐Square Tests comparing duck nest numbers during 2019, 2020, 2021, 2022, and 2023 with the expected value if nest abundance were evenly distributed across years for cinnamon teal (teal), mallard, and gadwall. Duck nest abundances were a count of all nests we found during a single year, correcting it the variability by the variability in daily survival rates (DSR) among years. We achieved this by dividing the number of nests found during a specific year by that year's DSR raised to the mean embryo age in days when the nest was first discovered (McPherson et al. 2003). For all variables, *d.f.* = 4. The same area was surveyed each year. Data were collected at US Bear River Migratory Bird Refuge, Utah.

Species	2019	2020	2021	2022	2023	Total	Expected value	*χ* ^2^	*p*
Teal	292	106	51	30	84	563	112.6	154.95	< 0.00001
Mallard	74	19	21	36	18	168	31.8	26.93	0.00002
Gadwall	97	36	46	7	34	220	44.0	48.89	< 0.00001
All species	463	161	118	73	136	951	190.2	194.47	< 0.00001

**TABLE 3:** Nest densities (numbers/km^2^) for cinnamon teal (teal), mallard, and gadwall at the US Bear River Migratory Bird Refuge, Utah during 2019, 2020, 2021, 2022, and 2023. Nest density was calculated by dividing nest abundance by the area searched (0.276 km^2^). The same area was surveyed each year.

Nest density	2019	2020	2021	2022	2023	$\bar{x}$
Teal nests	1058	384	185	109	304	409
Mallard nests	268	69	76	130	85	126
Gadwall nests	351	130	167	25	123	159
All duck nests	1677	583	428	264	493	694

The mean day when ducks started incubating their nests (incubation‐initiation date) differed among species (*F* = 6.15, *d.f*. = 2, 702, *p* = 0.002); teal started on mean ordinal day 159, mallards on day 160, and gadwalls started on day 163. Hence, we analyzed each duck species separately to determine the impact of years on incubation‐initiation date. We found that year had no effect on incubation‐initiation date for any duck species (Table 4).

**TABLE 4:** One‐Way Analysis of Variance (ANOVA) est comparing the ordinal day of incubation initiation (i.e., the day when incubation began and embryos started growing) to determine if incubation initiation days varied among years for cinnamon teal (teal), mallard, gadwall, all duck species combined nesting at US Bear River Migratory Bird Refuge, Utah. Values shown are means (± SD) of ordinal days.

Species	2019	2020	2021	2022	2023	Grand $\bar{x}$	*F*	*d.f*.	*p*
Teal	159 ± 12	159 ± 15	156 ± 10	157 ± 11	162 ± 10	159 ± 12	1.78	4, 420	0.13
Mallard	161 ± 13	153 ± 17	160 ± 6	160 ± 11	161 ± 12	160 ± 13	1.20	4, 116	0.31
Gadwall	164 ± 12	161 ± 16	164 ± 6	168 ± 7	162 ± 9	163 ± 12	0.73	4, 154	0.57
All species	160 ± 12	159 ± 16	160 ± 9	159 ± 11	162 ± 10	160 ± 12	1.22	4, 700	0.30

Clutch sizes differed among duck species (*F* = 10.63, *d.f*. = 2, 703, *p* = 0.0003) with gadwall having a larger clutch size ($\bar{x}$ = 8.6 eggs) than either teal (7.9) or mallard (7.2). Hence, we analyzed each duck species separately to determine the impact of years on clutch sizes. We found that clutch size differed among years for teal, mallard, gadwall, and all species combined (Table 5). Clutch sizes were smallest during the 2 dry years: 6.5 during 2021 and 6.8 during 2022 versus 8.4 during 2019, 7.9 during 2020, and 7.8 during 2023.

**TABLE 5:** One‐way analysis of variance (ANOVA) test comparing clutch sizes among years for cinnamon teal (teal), mallard, gadwall, all duck species combined nesting at US Bear River Migratory Bird Refuge, Utah. Values shown are means (± SD) of clutch sizes.

Species	2019	2020	2021	2022	2023	Grand $\bar{x}$	*F*	*d.f*.	*p*
Teal	8.3 ± 2.2	7.4 ± 2.6	6.5 ± 2.9	7.6 ± 2.6	7.8 ± 2.8	7.9 ± 2.5	4.66	4, 416	0.001
Mallard	8.1 ± 2.2	8.2 ± 2.4	5.4 ± 2.6	6.2 ± 2.4	6.2 ± 3.2	7.2 ± 2.6	5.56	4, 116	0.0004
Gadwall	8.9 ± 2.2	9.3 ± 2.2	6.9 ± 2.8	6.8 ± 1.9	8.5 ± 28	8.6 ± 2.5	4.60	4, 151	0.002
All species	8.4 ± 2.2	7.9 ± 2.6	6.5 ± 2.8	6.8 ± 2.5	7.8 ± 2.8	8.0 ± 2.5	10.94	4, 697	< 0.00001

We were able to determine the fate of 683 nests from 2019 through 2023. Across all years and all duck species, 386 (57%) nests were depredated, 51 (7%) were abandoned, and 246 (36%) were successful based on the apparent method. The number of nests that were depredated did not differ among duck species (*χ* ^2^ = 5.15, *d.f*. = 2, *p* = 0.08); nor did the number of nests that were abandoned (*χ* ^2^ = 2.30, *d.f*. = 2, *p* = 0.32) or successful (*χ* ^2^ = 5.10, *d.f*. = 2, *p* = 0.08). The number of nests that were depredated versus not depredated differed among years for all species combined and for each duck species when tested separately (Tables 6 and 7). The number of nests that were abandoned versus not abandoned differed among years for all species combined and for teal, but not for mallard and gadwall when tested separately. The number of nests that were successful versus not successful differed among years for all species combined and for each duck species when tested separately.

**TABLE 6:** Number of cinnamon teal (teal), mallard, and gadwall nests that were either depredated, abandoned, or successful (at least 1 egg hatched) each year at the US Bear River Migratory Bird Refuge based on the 683 nests where we were able to determine their fate. To determine if the percentage of nests that were depredated, abandoned, or successful varied among years, we used a Pearson's chi‐square test (*d.f.* = 4) to compare for each year the number of nests that were depredated versus not depredated, abandoned versus not abandoned, and successful versus not successful.

	2019	2020	2021	2022	2023	All years	*χ* ^2^	*p*
Teal depredated	89	78	16	17	26	226	73.04	< 0.00001
Teal abandoned	14	4	6	2	9	35	15.49	0.004
Teal successful	119	6	1	1	26	153	80.82	< 0.00001
Mallard depredated	26	12	9	23	9	79	18.54	0.001
Mallard abandoned	4	1	0	1	2	8	2.12	0.71
Mallard successful	24	3	1	1	4	33	16.86	0.002
Gadwall depredated	26	24	15	5	11	81	24.65	0.0006
Gadwall abandoned	1	2	2	0	3	8	5.77	0.22
Gadwall successful	43	4	3	0	10	60	30.78	< 0.00001
All species depredated	141	114	40	45	46	386	114.52	< 0.00001
All species abandoned	19	7	8	3	14	51	13.73	0.008
All species successful	186	13	5	2	40	246	126.70	< 0.00001

**TABLE 7:** Percentage of cinnamon teal (teal), mallard, and gadwall nests that were depredated, abandoned, or successful (at least one egg hatched) each year. The results were obtained by dividing the number of depredated nests each year (Table 6) by the total number of nests each year where we were able to determine their fate. We then repeated the same process for the number of nests that were abandoned and the number of nests that were successful. Data were collected at US Bear River Migratory Bird Refuge (Utah).

	2019	2020	2021	2022	2023
Teal nests depredated	40	89	70	85	42
Teal nests abandoned	6	5	26	10	15
Teal nests successful	54	6	4	5	43
Mallard nests depredated	48	75	90	92	60
Mallard nests abandoned	7	6	0	4	13
Mallard nests successful	44	19	10	4	27
Gadwall nests depredated	37	80	75	100	46
Gadwall nests abandoned	1	7	10	5	12
Gadwall nests successful	62	13	15	5	42
All species depredated	41	85	75	90	46
All species abandoned	6	5	15	6	14
All species successful	54	10	9	4	40

Abundance of skunks, raccoons, and all predators differed among years (Table 8). Skunk abundance hit a low of 0.02 (number photographed per bridge per night) during the heavy snowfall year (2023) and reached a peak of 0.40 during the drought year of 2021. Raccoon abundance (0.22) also were lowest in 2023 and peaked at 0.98 during the drought year of 2020.

**TABLE 8:** Pearson's chi‐square tests comparing annual indices of skunk abundance, raccoon abundance and all predator abundance during 2020, 2021, and 2023 with the expected value if predator abundances were evenly distributed across years. Predator abundance was a measure of the mean number of predators that were photographed nightly crossing a single bridge during a single night over a 21‐day period in May right before ducks started incubating their nests. There were 12 bridges along our transect, and one camera was positioned at each bridge. The results for each year show the total number of predators photographed each year divided by the number of camera days. For all variables, *d.f.* = 2. Data were collected at US Bear River Migratory Bird Refuge, Utah.

Indices of predator abundance	2020	2021	2023	Expected value	*χ* ^2^	*p*
Skunk abundance	0.34	0.40	0.02	0.27	69.51	< 0.00001
Raccoon abundance	0.98	0.79	0.22	0.66	77.97	< 0.00001
All predator abundance	1.35	1.20	0.27	0.94	121.09	< 0.00001

One hundred forty‐two predators were removed from BRMBR during this study, including 45 striped skunks and 97 raccoons. Seventeen predators were removed during 2019, 44 in 2020, 81, in 2021, 0 in 2022, and 0 in 2023. The number of predators removed each year varied among years (Table 9) due to whether the BRMBR staff decided to conduct predator control during the year, and if so, the amount of effort the refuge decided to put into predator control each year. For example, the refuge decided not to remove predators during 2022 and 2023 (personal communication, Jennifer Wright, BRMBR).

**TABLE 9:** Pearson's chi‐square tests comparing the number of skunks, raccoons and all predators removed from the US Bear River Migratory Bird Refuge each year with the expected value if numbers of predators removed were evenly distributed across years. For all variables, *d.f.* = 4. All predators were removed by the refuge staff. Data were provided by the refuge. Information on which months the predators were removed was not available.

Number removed	2019	2020	2021	2022	2023	Total	Expected value	*χ* ^2^	*p*
Skunks	15	6	24	0	0	45	9.0	26.92	0.00002
Raccoons	2	38	57	0	0	97	19.4	77.48	< 0.00001
All predators	17	44	81	0	0	142	28.4	88.31	< 0.00001

Annual variation in the number of nests of all duck species corrected for DSRs was positively correlated with the amount of rainfall during April and May of that same year (*r* = 0.89, *r* ^2^ = 0.78, *F*_1,3_ = 10.94, *p* = 0.04). None of the independent variables that we examined were correlated with clutch sizes.

Annual variation in percentages of nests depredated were negatively correlated with the level of snowfall during the prior winter (*r* = −0.95, *r* ^2^ = 0.91, *F*_1,3_ = 30.15, *p* = 0.01) and with raccoon abundance (*r* = −0.95, *r* ^2^ = 0.91, *F*_1,1_ = 30.15, *p* = 0.01). The yearly percentage of successful nests was positively correlated with snowfall (*r* = 0.99, *r* ^2^ = 0.98, *F*_1,3_ = 184.14, *p* = 0.0009) and negatively with abundance of all predators (*r* = −1.00, *r* ^2^ = 0.99, *F*_1,1_ = 133.12, *p* = 0.06). Snowfall was correlated with skunk abundance (*r* = −1.00, *r* ^2^ = 1.00, *F*_1,1_ = 50,236, *p* = 0.003) but not with raccoon abundance (*r* = −0.93, *r* ^2^ = 0.87, *F*_1,1_ = 6.67, *p* = 0.24) or abundance of all predators (*r* = −0.97, *r* ^2^ = 0.94, *F*_1,3_ = 114.77, *p* = 0.16) due to the small sample sizes.

## 5 Discussion

During the 5 years of our study, duck nest densities in BRMBR exhibited an episodic pattern across years with a 10‐fold change in numbers of cinnamon teal nests, a 4‐fold change in mallards, and a 3‐fold change in gadwalls between the highest and lowest densities. For cinnamon teal, mallards, gadwalls, and all species combined, nest numbers increased during wet years and decreased during droughts. Krapu, Klett, and Jorde (1983) noticed a similar pattern among mallard nest densities in the Prairie Pothole habitat of North Dakota. Mallard nest densities varied from 2 nests/km^2^ in 1977 to 9 nests/km^2^ in 1963 and were correlated with pond densities, which was itself related to precipitation (Krapu, Klett, and Jorde 1983; Johnson et al. 2005; Bartzen et al. 2017). However, our nest densities (694/km^2^) were much higher than those reported by Krapu, Klett, and Jorde (1983).

We found that the percentage of nests that were successful peaked in the same year when nest densities nests peaked. Such would occur if ducks could predict which years nest success would be high at BRMBR and when it would be better to nest elsewhere, but that seem unlikely to us. Another hypothesis for this pattern is predator swamping, which occurs when there are so many duck eggs in an area that predators can only consume a small percentage of them before becoming satiated (Descamps 2019). Eggs in the rest of the nests survived long enough to hatch, resulting in a high percentage of duck nests that were successful and high DSRs. We believe that this may have occurred during 2019, when duck nests were very abundant in BRMBR. A high percentage of duck nests were also successful during 2023, but the number of duck nests was not particularly high that year. Instead, deep snow covered the ground at BRMBR for several months during the prior winter. We believe that few skunks and raccoons survived the winter because we counted few of them during the following spring, and some of the photographed raccoons were emaciated. Hence, predator swamping may have occurred during 2023.

What characteristics ducks use to make the decision to nest in BRMBR or at a more northern nesting site in the United States or Canada is unclear. One hypothesis argues that nesting ducks avoid marshes where mammalian predators are abundant. However, Bell and Conover (2023a) discovered that duck nests were equally abundant along impoundments where skunks and raccoons were abundant or scarce. A second hypothesis states that a duck's decision of where to nest is based on vegetation cover, which would be greater during wet years and sparser during droughts. Mallards and other dabbling ducks prefer to nest in tall dense stands of grasses and legumes and in brushy vegetation with overhead concealment (Baldassarre 2014). Bell and Conover (2023a) examined nest site selection in ducks nesting in GSL marshes and found that cinnamon teal preferred to locate their nest where there is overhead cover capable of concealing the nest from an overhead view, but mallards and gadwall did not.

The climate extremes that occurred from 2019 through 2023 did not impact the timing of incubation initiation. Instead, the mean incubation‐initianion day occurred between ordinal day 159 and 163 every year despite large swings in climate. This constancy may cause problems for nesting ducks during future periods of climate extreme because they may not be able to adjust the timing of nesting to meet local conditions.

Mean clutch sizes of ducks ranged from 8.4 during the wet year of 2019 to 6.5 during the dry year of 2021. Ducks produce eggs that are large relative to the mass of the hen and are energetically expensive to create (Baldassarre 2014). Duck species obtain much of the food resources necessary for egg production after arriving on the breeding grounds (Jönsson 1997). For example, gadwall arrive on GSL marshes an average of 28 days prior to egg‐laying and use their time to forage locally (Gates 1962). This provides evidence to explain why we found that clutch sizes for all duck species were lowest during the two drought years (2021 and 2022) and highest during the 2 wet years (1999 and 2023). We would not have expected this pattern if food for egg production was obtained on the wintering grounds rather than locally. Like us, Krapu, Klett, and Jorde (1983) also reported a decline of 0.7 eggs in mallard clutches between a dry and wet year. During the egg‐laying period, female ducks exhibit a preference for protein, which they acquire from invertebrates (Baldassarre 2014). Among egg‐laying hens in North Dakota, 99% of the diet of blue‐winged teal (*Spatula discors*) consisted of invertebrates, as did 70% of mallards and 72% of gadwalls (Swanson, Krapu, and Serie 1979; Swanson, Meyer, and Adomaitis 1985). We hypothesize that the annual variation in clutch sizes among ducks nesting in GSL marshes may be correlated with local densities of aquatic invertebrates, but we lack the data to test this hypothesis.

It is unclear what happened to ducks that did not nest in BRMBR during the droughts. Foote (1989) may provide an answer to where these ducks went; he studied ducks nesting in GSL marshes before, during, and after the extreme flooding of GSL between 1983 and 1986. Consecutive years of heavy snowfall during those years caused water level of GSL to reach high enough to flood BRMBR and all other GSL marshes. Wave action destroyed the dams, and GSL's hypersaline water flowed into the previous freshwater marshes, killing aquatic vegetation. In response, ducks that had nested in GSL marshes abandoned them. Foote (1989) reported that the displaced ducks did not nest in other marshes in northern Utah; instead, the displaced ducks moved northward in the flyway to nest in Canada. During our study, ducks may have made the same decision to nest farther north during the 2 drought years. The BRMBR is located at the southern end of the breeding range for cinnamon teal, mallards, and gadwalls. We hypothesize that these ducks continued their northward migration after finding unsuitable nesting conditions in GSL marshes. The large numbers of ducks that nested in GSL marshes prior to the 1983–1984 floods have never returned despite the passage of 40 years (Bellrose 1980; Kear 2005; Baldassarre 2014; Olson 2016). Time will determine if the increasing frequency and intensity of extreme weather events caused by climate change will have a similar long‐term effect on the number of ducks nesting on GSL marshes.

The continental population of mallards averages 7.5 million and gadwalls 2.8 million (Baldassarre 2014). Only a small percentage of their population nest in GSL marshes; so what happens on GSL marshes has only a minor effect on their continental populations. This is not true for cinnamon teal, which is one of the least abundant duck species in North America. Its population is also one of the hardest to assess due to the similar appearance of blue‐winged teal and cinnamon teal hens and juveniles. For this reason, many population surveys lump cinnamon teal in with the much more abundant blue‐winged teal, making it difficult to assess the size of the cinnamon teal population (Baldassarre 2014). Breeding bird surveys should be more useful because male cinnamon teal in breeding plumage are easy to distinguish, but many of the breeding pairs of cinnamon teal that are spotted in GSL marshes migrate farther north to nest and may not reflect local breeding populations. Bellrose (1980) estimated that there were only 300,000 cinnamon teal in North America, with half of them nesting in GSL marshes prior to the 1980s. More recently, Olson (2016) reported 10,000 cinnamon teal nesting on Great Salt Lake marshes and the rest of Utah combined. We cannot speculate on the current size of the North American population of cinnamon teal, but we believe that there were < 10,000 cinnamon teal nests on GSL marshes during our studies (Bell and Conover 2023a, 2023b, 2023c). Our study supports the hypothesis that climate extremes caused by global warming will further decrease the numbers of ducks nesting in GSL marshes and the proportion of their nests that are successful. For cinnamon teal to lose half of its historic breeding range during the last 50 years is uncommon and concerning.

## Author Contributions

**Michael R. Conover:** conceptualization (lead), formal analysis (lead), funding acquisition (equal), project administration (lead), resources (equal), supervision (lead), validation (equal), visualization (equal), writing – original draft (lead), writing – review and editing (equal). **Mark E. Bell:** data curation (lead), investigation (equal), methodology (equal), writing – review and editing (equal).

## Ethics Statement

All work was conducted under Scientific Collector's permits from the Utah Division of Wildlife Resources (1BAND10069 and 2COLL10039) and the US Fish and Wildlife Service (MB693916‐0). Methods were approved by the Utah State University Institutional Animal Care and Use Committee (Protocol number: 10087).

## Conflicts of Interest

The authors declare no conflicts of interest.

## Data Availability

Data that support the findings of this study are openly available in Dryad at: https://doi.org/10.5061/dryad.9w0vt4bkh.
